# Adjuvant Alpha-Fetoprotein-Derived Peptide After Transarterial Chemoembolization in Patients With Hepatocellular Carcinoma: Protocol for a Safety Study

**DOI:** 10.2196/17082

**Published:** 2020-02-10

**Authors:** Akihiro Nomura, Takeshi Terashima, Eishiro Mizukoshi, Masaaki Kitahara, Toshinori Murayama, Shuichi Kaneko

**Affiliations:** 1 Innovative Clinical Research Center Kanazawa University Kanazawa Japan; 2 Department of Gastroenterology Kanazawa University Hospital Kanazawa Japan

**Keywords:** hepatocellular carcinoma, alpha-fetoprotein-derived peptides, safety trial

## Abstract

**Background:**

Hepatocellular carcinoma (HCC) is a worldwide health concern because of a continued increase in cases globally; furthermore, the prognosis for patients with HCC remains poor. Transarterial chemoembolization (TACE) has been established as the standard of care for the intermediate stage of HCC; however, no therapeutic agents are available to reduce the high rate of recurrence.

**Objective:**

This study aims to evaluate the safety of alpha-fetoprotein (AFP)-derived peptides for patients with HCC post-TACE.

**Methods:**

This will be an open-label, single-arm, multicenter study to evaluate the safety of AFP-derived peptides (AFP 357 and AFP 403), which contain histocompatibility antigen-A24-restricted cytotoxic T lymphocyte epitopes from tumor antigens expressed in HCC and is recognized at a high rate by lymphocytes in patients with HCC. Protocol treatment will consist of six courses of the subcutaneous administration of 3 mg each of AFP 357 and AFP 403. A total of 14 patients will be included in this study, the first 6 as a main analysis target group and an additional 8 as an extended cohort from three institutions in Japan. The primary endpoint will be the occurrence of serious adverse events (safety profile). The secondary endpoints will include time to progression, overall survival, completion rate, and adverse events (efficacy profile).

**Results:**

We have recruited 14 patients with HCC as of December 2019. The final follow-up will be completed by March 2020.

**Conclusions:**

In this study, we will evaluate the safety profile of AFP-derived peptides for patients with HCC post-TACE. We believe that this study will provide useful information and will help to design a subsequent phase II trial based on the results.

**Trial Registration:**

Japan Registry of Clinical Trials jRCTs041180155; https://jrct.niph.go.jp/latest-detail/jRCTs041180155

**International Registered Report Identifier (IRRID):**

DERR1-10.2196/17082

## Introduction

Hepatocellular carcinoma (HCC) is the sixth most common cancer and the third leading cause of cancer-related mortality worldwide [1]. Certain therapies are effective for treating different stages of HCC [2]. However, there is a high rate of metachronous and multifocal recurrence, even after curative treatment. Transarterial chemoembolization (TACE) has been established as the standard of care for the intermediate stage of HCC [3-6]. However, TACE is inferior to resection or percutaneous treatment in terms of curability and has a high rate of recurrence near the treated lesion, despite being judged effective based on posttreatment imaging studies. Moreover, patients with HCC are often in a cirrhotic state, in which the underlying liver has high carcinogenic potential, and relapse occurs at a high rate at sites remote from the treated lesion. Therefore, attempts are being made to develop therapies to prevent such recurrence. Sorafenib [7,8], brivanib [9], and olantinib [10] have been tested in combination with TACE in an adjuvant setting, but none of these trials have validated their use. Therefore, observation is the standard treatment strategy for patients with HCC post-TACE.

Since the identification of the melanoma antigen-encoding gene in 1991, the human immune system has been shown to recognize tumor antigens and eliminate tumor tissue. The primary cell type responsible for tumor cell clearance is the T cell, which recognizes a peptide fragment complex composed of a major histocompatibility antigen present on the surface of the tumor cell and a protein produced by the tumor cell and exhibits cytotoxic activity. Several tumor-specific antigens and peptides, along with their amino acid sequences, have been identified, and immunotherapy based on these peptides has been attempted. Some immunotherapy approaches have been able to induce T cells to attack tumor cells in humans, demonstrating antitumor effects.

We have screened and compared many candidates as targets for immunotherapy strategies in HCC [11-13]. Based on these findings, two alpha-fetoprotein (AFP)-derived peptides, AFP 357 and AFP 403, were identified as attractive compounds to activate cytotoxic T cells in patients with HCC, and preclinical and clinical studies were conducted using these molecules. In a phase I study, AFP 357 and AFP 403 were administered to 20 patients with advanced HCC. In all patients, no serious adverse events were observed. Moreover, complete response was obtained in 1 patient and tumor control achieved in 8 patients, which was according to the Response Evaluation Criteria in Solid Tumors (RECIST). The immunological effects of AFP-derived peptides were confirmed in 5 of 15 patients (33%), for whom efficacy was evaluated after 3 or more doses [11]. Based on these findings, immunotherapy using AFP-derived peptides might be a promising therapeutic strategy for patients with HCC post-TACE as it is suggested for patients with more advanced HCC. The aim of this study, therefore, was to evaluate the safety of AFP-derived peptides for patients with HCC post-TACE.

## Methods

### Overall Study Design

**Figure 1:**
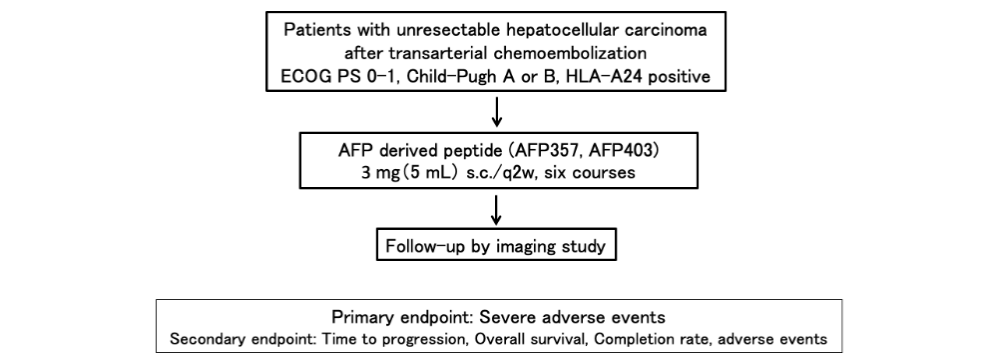
Diagram of study design. ECOG: Eastern Cooperative Oncology Group; PS: performance status; HLA: human leukocyte antigen; AFP: alpha-fetoprotein.

This will be an open-label, single-arm, multicenter study (Figure 1). The primary endpoint of this study will be serious adverse events (safety profile); the secondary endpoint will include time to progression, overall survival, completion rate, and adverse events (efficacy profile). This study will be conducted in accordance with the Declaration of Helsinki, Clinical Trials Act, Ethical Guidelines for Medical and Health Research Involving Human Subjects, and all other applicable laws and guidelines in Japan. The protocol of this study was approved by the Institutional Review Board at Kanazawa University Hospital and is registered at the Japan Registry of Clinical Trials (jRCTs041180155).

### Study Participants

We will first recruit 14 patients at Toyama City Hospital and Fukui-ken Saiseikai Hospital, subsequently adding those at the Kanazawa University Hospital, from October 2017 to December 2019. All patients will meet the inclusion criteria and no exclusion criteria will be applied (Textbox 1). Patients will be provided with comprehensive information about AFP-derived peptides and will provide written informed consent to participate in this study.

**Inclusion and exclusion criteria of this study.**

**Inclusion criteria**

1. The patient was clinically diagnosed with hepatocellular carcinoma (based on histology or imaging)
2. Transarterial chemoembolization was performed because resection or percutaneous local treatment was not indicated due to multiple occurrences
3. It was confirmed that good embolic effects were obtained for nodules treated with transarterial chemoembolization
4. Patients whose adverse events associated with Transarterial chemoembolization have resolved or not worsened
5. Patients with human leukocyte antigen–A24-positive human tumor histocompatibility antigen
6. Child-Pugh classification is A or B
7. Age at entry (full age) is 20 years or higher
8. The Eastern Cooperative Oncology Group performance status is less than or equal to 2 (0–2)
9. The most recent test value within 14 days prior to enrollment satisfies all of the following: neutrophil count≥1000/mm3, hemoglobin level≥8.0 g/dL, platelet count≥40,000/mm3, total bilirubin level ≤3.0 mg/dL
10. The patient has provided written consent to participate in the trial

**Exclusion criteria**

1. The patient has refractory ascites and moderate or severe pleural effusion
2. The patient had a history of hepatic encephalopathy within 3 months before registration
3. Esophagogastric varices at risk of bleeding have been identified and no preventive measures have been taken
4. Active malignancy
5. Blood transfusions, blood products (containing a preparation of albumin), and blood-enhancing factor products such as granulocyte colony-stimulating factor administered within 2 weeks prior to enrollment
6. The patient received continuous systemic administration of steroids or other immunosuppressants (oral or intravenous administration)
7. Serious complications (including heart failure, renal failure, hepatic failure, bleeding peptic ulcer, intestinal paralysis, intestinal obstruction, and poorly controlled diabetes mellitus)
8. Infection (except for viral hepatitis) that requires systemic treatment
9. A woman who is pregnant, possibly pregnant, within 28 days after childbirth, or breastfeeding; a man who wishes to become pregnant with his partner
10. The patient is considered to have a psychiatric disorder or psychiatric symptom, and it is difficult for them to participate in the study
11. The patient has serious hypersensitivity to alpha-fetoprotein-derived peptides or components of adjuvants
12. Neither computed tomography nor magnetic resonance imaging with contrast agent can be performed due to drug allergy
13. The attending physician determines that participation in this study is inappropriate

### Intervention

The treatment drug will be AFP-derived peptides (AFP 357 and AFP 403), which contain human leukocyte antigen (HLA)-A24–restricted cytotoxic T lymphocyte epitopes derived from tumor antigens expressed in HCC that are recognized by lymphocytes in patients with HCC at a high rate [11,13]. The AFP-derived peptides used in this study will be produced by Neo MPS Inc (San Diego, California) at Good Manufacturing Practice grade. One course of treatment will consist of the subcutaneous administration of 3 mg each of AFP 357 and AFP 403 on day 1 followed by 13 days of rest. Treatment will be repeated for up to six courses unless the following discontinuation criteria are applicable: obvious tumor progression based on radiological or physical findings, any adverse event that causes the discontinuation of protocol therapy, patient refusal, or death during protocol therapy. Before the treatment course starts, we will confirm that no grade 2 or higher adverse events exist. The administration of anticancer drugs other than AFP 357 and AFP 403 and procedures such as TACE, radiotherapy, hormonal therapy, or immunotherapy will not be permitted during the study. However, corticosteroids needed for fatigue; anorexia and emaciation; diuretics; amino acid preparations for ascites; hepatic edema; treatment of complications such as hypertension or diabetes mellitus; and antiemetics for nausea or vomiting will be permitted.

### Follow-up Schedule

Table 1 shows the overall follow-up schedule. In this study, six courses of AFP-derived peptide administration are defined as the protocol treatment, and the entire study period is classified into the following three periods: pretreatment period, from consent to the start of treatment; treatment period, from the start of treatment to 30 days after the last administration (to assess adverse events related to protocol treatment); and follow-up period, from the end of the treatment period to death or to the final follow-up of this study. Before registration, hepatitis virus markers including hepatitis B virus antigen (HBsAg) and hepatitis C antibody will be assessed. Hepatitis B virus-DNA will be checked in positive HBsAg results. Hepatitis B surface antibody and Hepatitis B core antibody will be checked if HBsAg results are found to be negative. Chest x-ray, electrocardiogram, and tumor markers including AFP and des-carboxy-prothrombin will be assessed prior to TACE treatment. Patient’s general condition will also be evaluated using Eastern Cooperative Oncology Group (ECOG) performance status, body weight, encephalopathy, blood tests (complete blood count, blood chemistry, and coagulation), objective findings, contrast-enhanced computed tomography, and tumor markers within 14 days prior to registration.

**Table 1:** Follow-up schedule for trial protocol.

Assesments		Pretreatment period		Treatment period					Follow-up period
					During protocol treatment		Postprotocol treatment		
**General condition**									
	Adverse events		14 days prior^a^		Date of administration^b^		1 month after^c^		N/A^d^
	Height		Before registration		N/A		N/A		N/A
	Body weight		14 days prior		N/A		N/A		N/A
	ECOG^e^ performance status		14 days prior		Date of administration		1 month after		N/A
**Clinical examination**									
	WBC^f^ (differential), hemoglobin, platelets		14 days prior		As needed		As needed		N/A
	Albumin, bilirubin, aspartate transaminase, alanine transaminase, BUN,^g^ creatinine, lactate dehydrogenase, alkaline phosphatase, CRP,^h^ PT,^i^ PT-INR,^j^ PT activity levels		14 days prior		As needed		As needed		N/A
	HBs^k^ antigen, HCV^l^ antibody		Before registration		N/A		N/A		N/A
	HBs antibody, HBc^m^ antibody, HBV^n^-DNA		As needed		As needed		N/A		N/A
	Alpha-fetoprotein, PIVKA-II^o^		14 days prior		12 weeks after^p^		N/A		12 weeks with no progression^q^
	Contrast-enhanced computed tomography		14 days prior		12 weeks after		N/A		12 weeks with no progression
	Arterial blood gas		N/A		As needed		N/A		N/A

^a^Within 14 days prior to registration.

^b^Date of administration or the day before administration.

^c^1 month after discontinuation of protocol therapy or until the start of posttreatment.

^d^Not applicable.

^e^ECOG: Eastern Cooperative Oncology Group.

^f^WBC: white blood cell.

^g^BUN: blood urea nitrogen.

^h^CRP: C-reactive protein.

^i^PT: prothrombin time.

^j^INR: international normalized ratio.

^k^HBs: hepatitis B surface.

^l^HCV^:^ hepatitis C virus.

^m^HBc: hepatitis B core.

^n^HBV: hepatitis B virus.

^o^PIVKA-II: protein induced by vitamin K absence or antagonist-II.

^p^12 weeks after enrollment (allow ± 2 weeks of change).

^q^12 weeks after enrollment in cases of no apparent disease progression.

During the treatment period, patients’ general condition including ECOG performance status, encephalopathy, and adverse events will be evaluated at every visit for the safety profile. Blood tests, chest X-ray, or electrocardiogram will also be performed if needed. Contrast-enhanced computer topography (CT) and tumor markers will be evaluated for the efficacy endpoint every 12 weeks after enrollment. Contrast-enhanced CT and tumor markers will also be evaluated until tumor progression is confirmed during the follow-up period.

### Endpoints

The primary endpoint of this study will be the occurrence of serious adverse events (safety profile) during the treatment protocol. Serious adverse events are defined as any adverse events causing death, life-threatening condition, hospitalization (initial or prolonged), disability or permanent damage, or congenital anomaly/birth defect outcomes. Adverse events will be evaluated according to the Common Terminology Criteria for Adverse Events v 4.0 JCOG Version (Japanese translation of the NCI-Common Terminology Criteria for Adverse Events v 4.0) (CTCAE v 4.0 - JCOG). Each adverse event will be graded based on the definitions of grades 0-4. If a specific procedure is described based on grade, it will be graded for its clinical need. For example, if a patient has increased pleural effusions and oxygen or chest drainage is indicated, the patient may refuse. In such cases, the grading will be based on the medical judgment of what should have been performed rather than whether treatment was actually administered.

The secondary endpoints will be the efficacy profile including time to progression, overall survival, and completion rate as well as adverse events. The efficacy profile will be evaluated according to the Revised RECIST guidelines for the evaluation of the therapeutic efficacy of solid tumors Version 1.1 every 12±2 weeks after registration. The efficacy will be evaluated based on the same examination conditions as the baseline evaluation, such as slice width if imaging results are available. However, plain radiography and contrast studies with different modalities will be acceptable if a contrast allergy is detected prematurely and testing with the same modality cannot be continued.

### Data Monitoring

Selected monitoring staff will conduct centralized data monitoring. The trial database will be monitored and reviewed annually by the selected monitoring staff, and data queries will be raised if necessary.

### Sample Size

We first calculated the sample size for this study based on our in-house data on patients with HCC. Kanazawa University Hospital conducted TACE for 350 patients with HCC from January 2005 to December 2011. In this group of Japanese patients with HCC, approximately 60% of the individuals were HLA–A24-positive, and approximately 30% of the candidates were expected to drop out based on eligibility criteria and exclusion criteria. Considering that this is a safety confirmation study, the rate of obtaining consent for this study was suggested to be 30%, and as such, approximately 8 patients were expected to be recruited annually. On January 2019, the safety profiles of the 6 cases included in this study were reviewed by authorities of the Ministry of Health, Labour and Welfare, and the safety of the treatment protocol was confirmed. Based the advice of the Efficacy and Safety Committee, the 6 cases were designed as the main analysis target group for the safety confirmation part. Registration of additional cases continued at Fukui-ken Saiseikai Hospital, Toyama City Hospital, and Kanazawa University Hospital until December 31, 2019. The target number of patients in this extended cohort was set at 8 based on the expected number of patients at the cooperative research institution. In total, we included 14 patients from three hospitals.

### Statistical Analysis

Safety profiles will be summarized using appropriate descriptive statistical methods. If necessary, we will obtain an accurate 95% confidence interval based on the binomial distribution. Time to progression is defined as the period from the date of enrollment to the date of evaluation of tumor progression. Tumor progression includes both “progressive disease” based on imaging examination, evaluated according to RECIST version 1.1 (radiological progression), and tumor progression that cannot be confirmed by imaging examination (clinical progression). The date of the tumor progression evaluation will be determined by when imaging examination is performed for radiological progression and clinical judgement is made for clinical progression. Overall survival is defined as the period from the date of enrollment to the date of death from any cause. Cumulative survival will be estimated using the Kaplan-Meier method. Completion rate will be calculated as the number of patients receiving six courses of AFP-derived peptide divided by the number of patients enrolled.

## Results

We recruited a total of 14 patients with HCC and ended recruitment in December 2019. The final follow-up will be completed in March 2020. Then, we will perform data analysis and disseminate the study results in late 2020.

## Discussion

We previously reported the safety and efficacy of these AFP-derived peptides for patients with HCC who were intolerant or refractory to standard therapy, or in other words, those with advanced-stage HCC [11]. Recently, numerous agents have been developed and subjected to ongoing clinical trials for such patients, and some of them were found to prolong survival in large phase III trials [14-16]. However, no agents have proven their ability to suppress metachronous and multifocal recurrence in patients with HCC post-TACE [7-10]. Due to the large number of patients experiencing recurrence, there is a need for further research.

As another rationale for the target population, we previously found that more advanced-stage patients harbor more antitumor suppressor cells [17,18], which suggested that it was better to select patients at an earlier stage to maintain their antitumor immunity and maximize the efficacy of cancer peptide therapy. Moreover, earlier-stage patients are considered suitable in terms of feasibility and safety, because patients with more advanced-stage HCC tend to experience deterioration of hepatic reserve or general condition [19]. With these considerations, we set the target population as patients post-TACE administration for this study.

The primary aim of this study is to evaluate the safety profile of AFP-derived peptides, namely AFP 357 and AFP 403, for patients with HCC post-TACE. We will also evaluate feasibility and efficacy endpoints to obtain helpful information for the design of a subsequent phase II trial. We believe that this study will show the safety of AFP-derived peptides for patients with HCC post-TACE, and we plan to design the subsequent phase II trial based on its results.

## Abbreviations

AFP: alpha-fetoprotein
CT: computer topography
ECOG: Eastern Cooperative Oncology Group
HBsAg: hepatitis B virus antigen
HCC: hepatocellular carcinoma
HLA: human leukocyte antigen
RECIST: Response Evaluation Criteria in Solid Tumors
TACE: transarterial chemoembolization.
